# Scan-rescan reliability assessment of brain volumetric analysis across scanners and software solutions

**DOI:** 10.1038/s41598-025-15283-3

**Published:** 2025-08-14

**Authors:** Eva Bürkle, Ahmad Nazzal, Alexander Debolski, Ulrike Ernemann, Tobias Lindig, Benjamin Bender

**Affiliations:** 1 Department of Diagnostic and Interventional Neuroradiology, University Hospital Tübingen, Hoppe-Seyler-Straße 3, 72076 Tübingen, Germany; 2 AIRAmed GmbH, Konrad-Adenauer-Str. 13, 72072 Tübingen, Germany; 3 High-Field MR Center, Max Planck Institute for Biological Cybernetics, Tübingen, Germany; 4 Department of Neurosurgery, University Hospital Tübingen, Tübingen, Germany

**Keywords:** Computational neuroscience, Data processing, Machine learning, Nervous system, Computational science, Scientific data, Software, Neurological disorders

## Abstract

Automated brain volumetry shows promise in improving the screening and monitoring of neurodegenerative diseases. However, the reliability of measurements across different scanners and software remains uncertain. This study assessed the reliability of gray matter, white matter, and total brain volume measurements from seven volumetry tools, using six scanners across two scanning sessions, performed within 2 h the same day, in twelve subjects. Generalised estimating equations models showed significant effects of both software and scanner on all measurements with stronger effect of software (*p* < 0.001). Percentage of coefficient of variation (CV) was calculated to measure scan-rescan reliability. Median CV across scanners of AssemblyNet and AIRAscore was less than 0.2% for grey and white matter, and 0.09% for total brain volume; while FreeSurfer, FastSurfer, syngo.via, SPM12, and Vol2Brain had a CV greater than 0.2%. In Bland-Altman analysis there was no systematic difference, but limits of agreement differed greatly between methods. Based on these findings, we recommend using the same scanner and software combination across sessions to ensure that observed changes in brain volume are reliable and clinically valuable.

**Supplementary Information:**

The online version contains supplementary material available at 10.1038/s41598-025-15283-3.

## Introduction

With recent advancements in artificial intelligence (AI), an increasing number of brain volumetric tools, including certified as medical devices, are available on the market^1–3^. Brain volumetric analyses are promising tools for quantifying brain volume loss in neurodegenerative diseases. For instance, they can be used to assess Alzheimer and other dementias and subtypes of Parkinson’s syndromes^4–7^ and monitor brain and spinal cord atrophy in multiple sclerosis to predict clinical outcomes and monitor therapy response^8^. For the results of automated volumetry to be of clinical value, it is important to understand the scan-rescan reliability of measurements^9^. Several factors affect the reliability of volumetric measurements: first, subject movement during the scan; second, the scanner’s intrinsic signal-to-noise ratio and inhomogeneities of the B0 and B1 fields, including differences in field strength^10^; third, the sequence parameters affecting the contrast-to-noise ratio between grey and white matter^11^; and fourth, the segmentation algorithm used^7^. This impacts both scientific research and clinical results when comparing measurements from temporally spaced volumetric examinations^4–6^. Therefore, there is a need to investigate the reliability of volumetric software in regards of reproducibility of measurements. Several studies have compared research brain volumetric tools in regards of their test-retest performance^7,8^. Nevertheless, knowledge about the performance of certified medical devices and new scientific AI based segmentation tools is scarce. Therefore, this study aims to systematically investigate the effect of different scanners of the same vendor using seven different segmentation algorithms including certified medical device software, new well performing AI based tools and established scientific tools on brain volumetric measurements (e.g. FreeSurfer, which is still one of the most widely used volumetric research-tools^12^.

## Results

### Demographics

Twelve healthy subjects (6 women, 6 men) with a mean age of 35.3 years (± 8.5 years) were examined between March 2021 and November 2021.

### General estimation equations results

#### Effect of software and scanner on measured Gray matter volume

In the analysis of the effect of session, scanner, and software on gray matter (GM) volume measurement, significant main effects were observed for software (Wald χ² = 22377.50, df = 6, *p* < 0.001) and scanner (Wald χ² = 91.76, df = 5, *p* < 0.001) but not for session (Wald χ² = 1.47, df = 1, *p* = 0.23) – see Table 1. The interaction between session and software was not statistically significant (Wald χ² = 2.10, df = 5, *p* = 0.834) but a significant interaction was found for session and scanner (Wald χ² = 30.46, df = 6, *p* < 0.001). However, post-hoc analysis showed that only the interaction between session and Vida scanner was significant (Wald χ² = 4.224, df = 1, *p* = 0.040), which is most likely an alpha-error. Moreover, the interaction between scanner and software was significant (Wald χ² = 1.279 × 10¹², df = 13, *p* < 0.001). Specifically, interaction between Aera scanner and AIRAscore software (Wald χ² = 265.229, df = 1, *p* < 0.001), FastSurfer software (Wald χ² = 5.465, df = 1, *p* = 0.019), FreeSurfer software (Wald χ² = 35.167, df = 1, *p* < 0.001); Aera3 scanner and AIRAscore software (Wald χ² = 261.100, df = 1, *p* < 0.001), FastSurfer software (Wald χ² = 4.596, df = 1, *p* = 0.032), FreeSurfer software (Wald χ² = 10.623, df = 1, *p* = 0.001); Avanto scanner and AIRAscore software (Wald χ² = 293.339, df = 1, *p* < 0.001), FastSurfer software (Wald χ² = 23.571, df = 1, *p* < 0.001), FreeSurfer software (Wald χ² = 32.964, df = 1, *p* < 0.001); Vida scanner and AIRAscore software (Wald χ² = 38.278, df = 1, *p* < 0.001), FastSurfer software (Wald χ² = 17.227, df = 1, *p* < 0.001); Vidafit scanner and AIRAscore software (Wald χ² = 233.987, df = 1, *p* < 0.001), FastSurfer software (Wald χ² = 7.889, df = 1, *p* = 0.005), FreeSurfer software (Wald χ² = 13.692, df = 1, *p* < 0.001), SPM12 software (Wald χ² = 21.262, df = 1, *p* < 0.001), syngo.via software (Wald χ² = 16.381, df = 1, *p* < 0.001), and Vol2Brain (Wald χ² = 11.859, df = 1, *p* < 0.001). Furthermore, the three-way interaction among session, scanner, and software (Wald χ² = 1.445 × 10¹², df = 15, *p* < 0.001) was also significant.

**Table 1:** Post-hoc analysis for constant terms of software and scanner Gray matter volume measurements with AssemblyNet and Prisma as reference. The table shows the parameters (Param), regression coefficient B (RCB), standard error (SE), 95% Wald confidence interval lower bound (Lower Int), 95% Wald confidence interval upper bound (Upper Int), Wald Chi-Square (Wald χ²), degrees of freedom (df), and significance level (sig).

Param.	RCB	SE	Lower Int	Upper Int	Wald χ²	df	sig
(Constant term)	777.822	16.6248	745.238	810.406	2189.006	1	< 0.001
Software							
AIRAscore	−93.271	7.6877	−108.339	−78.204	−147.197	1	< 0.001
FastSurfer	−119.480	6.6174	−157.028	−132.852	552.297	1	< 0.001
FreeSurfer	−119.480	6.3322	−131.891	−107.069	356.022	1	< 0.001
SPM12	−63.199	7.1135	−77.141	−49.257	78.931	1	< 0.001
Syngo.via	−63.848	7.3131	−78.182	−49.515	76.224	1	< 0.001
Vol2Brain	−67.408	5.8004	−78.776	−56.039	135.051	1	< 0.001
Scanner							
Aera	−3.075	2.5115	−7.998	1.847	1.499	1	0.221
Aera3	−1.567	1.8864	−5.264	2.131	0.690	1	0.406
Avanto	−7.190	2.3394	−11.775	−2.605	9.445	1	0.002
Vida	−11.567	2.8992	−17.249	−5.885	15.917	1	< 0.001
VidaFit	0.705	0.7800	−0.823	2.234	0.818	1	0.366

#### Effect of software and scanners on measured white matter volume

In the analysis of the effect of session, scanner, and software on white matter (WM) volume measurement, significant main effects were observed for both software (Wald χ² = 2218.32, df = 6, *p* < 0.001) and scanner (Wald χ² = 255.22, df = 5, *p* < 0.001) but not for session (Wald χ² = 0.78, df = 1, *p* = 0.38) – see Table 2. The interaction between session and scanner was not statistically significant (Wald χ² = 9.00, df = 5, *p* = 0.109). Weak significant interaction was found between session and software (Wald χ² = 16.91, df = 6, *p* = 0.01). However, post-hoc analysis showed no significant interaction between any software and session. Moreover, the two-way interaction between scanner and software was significant (Wald χ² = 1.376 × 10¹², df = 12, *p* < 0.001). Specifically, the interactions between Aera scanner and AIRAscore software (Wald χ² = 111.928, df = 1, *p* < 0.001), FastSurfer (Wald χ² = 15.697, df = 1, *p* < 0.001), and FreeSurfer (Wald χ² = 14.240, df = 1, *p* < 0.001); Aera3 scanner and FreeSurfer (Wald χ² = 20.801, df = 1, *p* < 0.001); Avanto scanner and AIRAscore software (Wald χ² = 130.165, df = 1, *p* < 0.001), FastSurfer software (Wald χ² = 36.870, df = 1, *p* < 0.001), FreeSurfer software (Wald χ² = 34.647, df = 1, *p* < 0.001), syngo.via software (Wald χ² = 5.167, df = 1, *p* = 0.023), Vol2Brain software (Wald χ² = 4.712, df = 1, *p* = 0.030); Vida scanner and AIRAscore software (Wald χ² = 69.986, df = 1, *p* < 0.001), FastSurfer software (Wald χ² = 20.466, df = 1, *p* < 0.001), FreeSurfer software (Wald χ² = 58.623, df = 1, *p* < 0.001), syngo.via software (Wald χ² = 7.710, df = 1, *p* = 0.005); Vidafit scanner and AIRAscore software (Wald χ² = 65.878, df = 1, *p* < 0.001), SPM12 software (Wald χ² = 9.629, df = 1, *p* = 0.002), syngo.via software (Wald χ² = 26.540, df = 1, *p* < 0.001). The three-way interaction among session, scanner, and software (Wald χ² = 55944.44, df = 11, *p* < 0.001) was also significant.

**Table 2:** Post-hoc analysis for constant terms of software and scanner white matter volume measurements and AssemblyNet and Prisma as reference. The table shows the parameters (Param), regression coefficient B (RCB), standard error (SE), 95% Wald confidence interval lower bound (Lower Int), 95% Wald confidence interval upper bound (Upper Int), Wald Chi-Square (Wald χ²), degrees of freedom (df), and significance level (sig).

Param.	RCB	SE	Lower Int	Upper Int	Wald χ²	df	sig
(Constant term)	462.426	10.1317	442.569	482.284	2083.151	1	< 0.001
Software							
AIRAscore	60.073	5.3362	49.614	70.532	126.732	1	< 0.001
FastSurfer	73.648	6.7092	60.498	86.798	120.498	1	< 0.001
FreeSurfer	70.948	6.3907	58.422	83.474	123.249	1	< 0.001
SPM12	−10.951	5.3258	−21.390	−0.513	4.228	1	0.040
Syngo.via	−20.222	5.5472	−31.094	−9.350	13.289	1	< 0.001
Vol2Brain	47.713	5.9353	35.539	58.805	63.165	1	< 0.001
Scanner							
Aera	13.792	1.3954	11.058	16.527	97.704	1	< 0.001
Aera3	14.501	1.1733	12.202	16.801	152.765	1	< 0.001
Avanto	10.483	1.4857	7.571	13.395	49.788	1	< 0.001
Vida	−1.893	1.5885	−5.007	1.220	1.420	1	0.233
VidaFit	0.148	0.8065	−1.433	1.729	0.034	1	0.854

#### Reproducibility of measurement for Gray matter volume, white matter volume and total brain volume

**Fig. 1:**
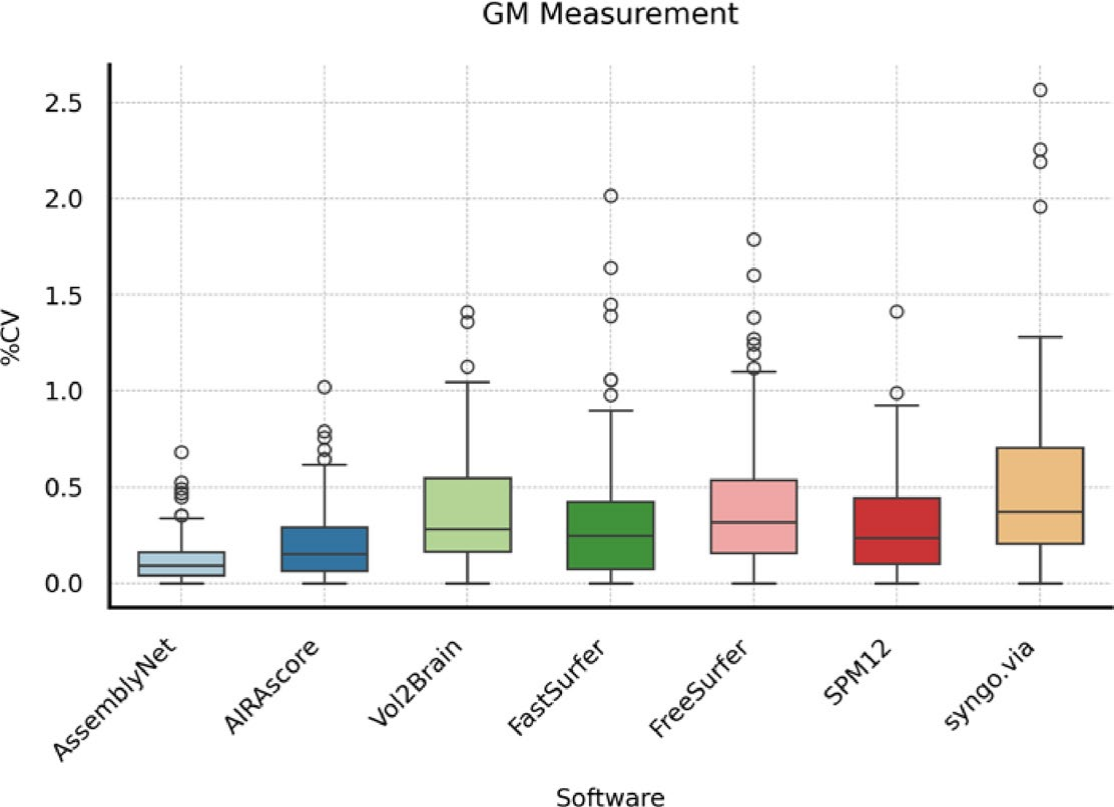
Boxplots showing the coefficient of variation (CV) for gray matter (GM) measurements across different software solutions. The horizontal line in each box represents the median, the box indicates the interquartile range (IQR), and circles represent individual outliers.

For GM volume measurements Vol2Brain, FastSurfer, FreeSurfer, SPM12, and syngo.via had a median CV of less than 1%. Only AssemblyNet and AIRAscore reached a median CV and an IQR of less than 0.2% (see Table 3; Fig. 1).

**Table 3:** Descriptive statistics for CV GM measurement.

Software	Median	25th Percentile	75th Percentile
AssemblyNet	0.08%	0.05%	0.18%
AIRAscore	0.13%	0.09%	0.27%
Vol2Brain	0.28%	0.17%	0.56%
FastSurfer	0.21%	0.14%	0.43%
FreeSurfer	0.31%	0.18%	0.56%
SPM12	0.27%	0.14%	0.45%
syngo.via	0.37%	0.23%	0.75%

**Fig. 2:**
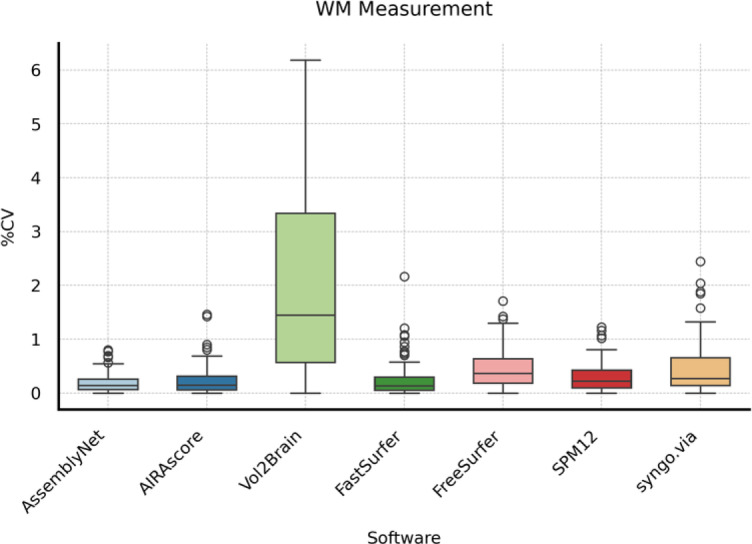
Boxplots showing the coefficient of variation (CV) for white matter (WM) measurements across different software solutions. The horizontal line in each box represents the median, the box indicates the interquartile range (IQR), and circles represent individual outliers.

For WM volume measurements, AssemblyNet, AIRAscore, and FastSurfer showed a median CV of less than 0.2%, while the first two outperformed FastSurfer on the IQR. Vol2Brain, SPM12, and syngo.via achieved a median CV between 0.23% and 0.37%. Vol2Brain showed a considerably lower performance with a median CV of 1.7% (see Table 4; Fig. 2).

**Table 4:** Descriptive statistics for CV WM measurement.

Software	Median	25th Percentile	75th Percentile
AssemblyNet	0.16%	0.08%	0.28%
AIRAscore	0.14%	0.09%	0.32%
Vol2Brain	1.70%	0.76%	3.04%
FastSurfer	0.15%	0.06%	0.37%
FreeSurfer	0.37%	0.19%	0.66%
SPM12	0.23%	0.13%	0.44%
syngo.via	0.29%	0.17%	0.63%

**Fig. 3:**
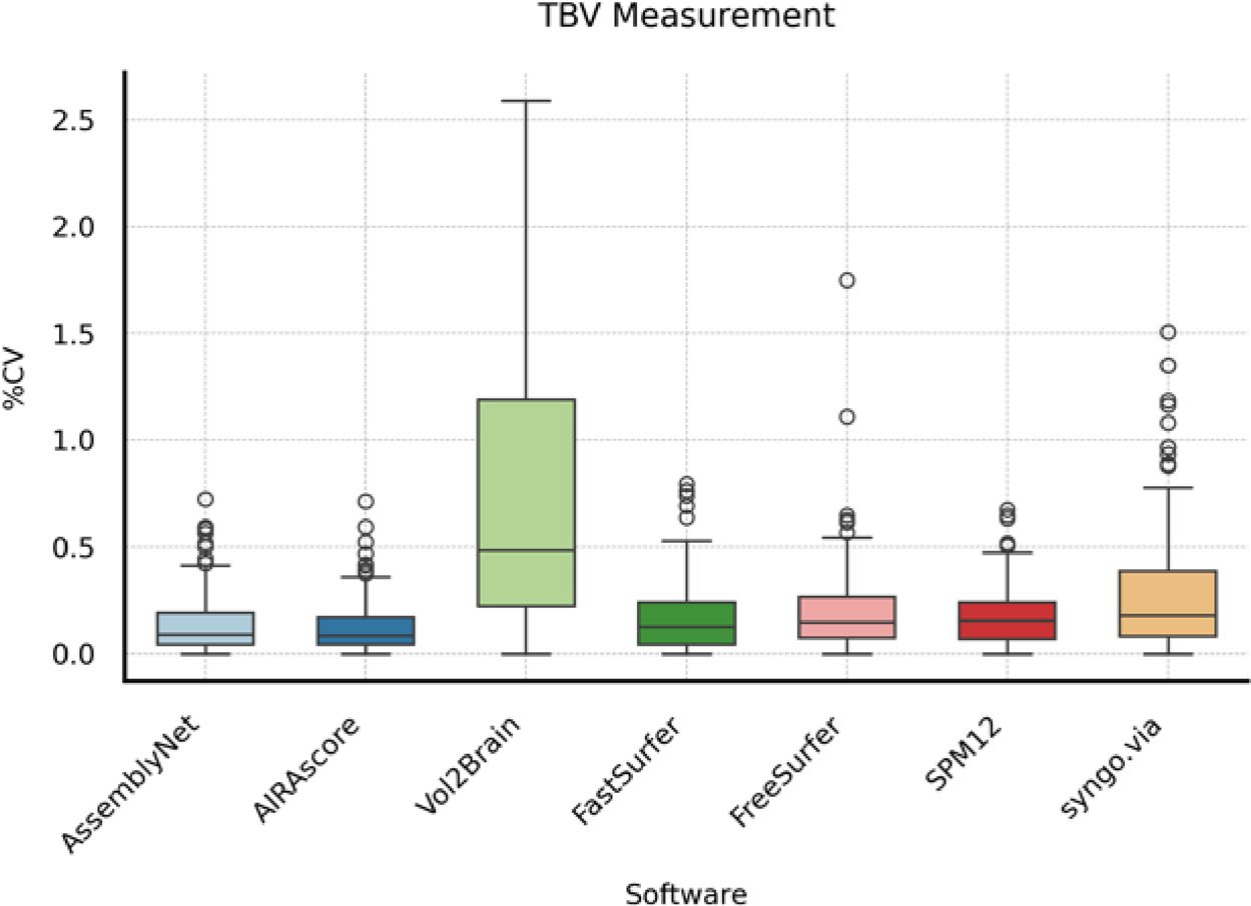
Boxplots showing the coefficient of variation (CV) for total brain volume (TBV) measurements across different software solutions. The horizontal line in each box represents the median, the box indicates the interquartile range (IQR), and circles represent individual outliers.

Total brain volume (TBV), as the largest volume and with an intrinsic error tolerance to GM versus WM misclassifications, showed as expected the smallest CV of the three evaluations. For TBV, AssemblyNet and AIRAscore outperformed the other software solutions with a median CV less than 0.1% and an IQR less than 0.2%. FastSurfer, FreeSurfer and SPM12 had a CV less than 0.2% and an IQR less than or around 0.2%. Syngo.via resulted in CV of 0.2% but with an IQR of around 0.3%, while Vol2Brain was most affected by the difference in WM volume estimates between measurements and showed the lowest performance (see Table 5; Fig. 3).

**Table 5:** Descriptive statistics for CV TBV measurement.

Software	Median	25th Percentile	75th Percentile
AssemblyNet	0.09%	0.04%	0.19%
AIRAscore	0.09%	0.05%	0.18%
Vol2Brain	0.62%	0.31%	1.04%
FastSurfer	0.14%	0.07%	0.26%
FreeSurfer	0.14%	0.08%	0.29%
SPM12	0.16%	0.09%	0.25%
syngo.via	0.20%	0.09%	0.42%

Bland-Altman-Plots with individual subjects and scanners revealed no systematic deviations of individual scanners and subjects or a systematic influence of the size of the measured volume on the difference, while the limits of agreement showed a similar effect as the CV for different software solutions (see supplemental material).

## Discussion

This study assessed the scan-rescan reliability of seven brain volumetric software solutions by examining within-day scans across six different scanners. Firstly, scanner, software and session effects were examined with generalized estimation equations (GEE). As observed in previous studies, the software had the largest impact on measured volumes showing significant variations in measured volumes^13–15^. Additionally, while the scanner was a relevant factor, its effect was lesser than the software, underpinning the relevance of using the same setup –scanner, software, and sequences– for performing follow-up examinations^14^. In terms of reliability, AssemblyNet and AIRAscore showed the lowest measurement error between scanning sessions using the same scanner, achieving a median CV of less than 0.09% TBV. Thus, the CV for both solutions falls within the range of TBV changes observed in healthy middle-aged subjects and is below the annual decline of 0.5% and 1% seen in multiple sclerosis (MS) patients of the same age group^15^. Furthermore, since total brain atrophy correlates with cognitive impairment in MS patients^8,16^, quantitative TBV measurements during therapy could help identify patients at risk, considering measurement error. Moreover, a previous study concluded that a CV below 2% is desirable in Alzheimer’s disease patients^9^. All tools but Vol2Brain had a 75th percentile CV of below 2% for gray matter and white matter – suggesting clinical utility in neurodegenerative diseases. Nevertheless, measurement error should be as small as possible to allow for detection of pathologic volume changes even in shorter follow-up periods.

In this study, we used six Siemens scanners. We selected the Siemens Prisma scanner as the “reference” due to its widespread use in research and high signal to noise ratio. This approach allowed us to efficiently manage the initial exploration of scanner/software interactions and provide results for any scanner/software combination, assessing their impact on the final volumetric result. However, deep learning models like AssemblyNet trained solely on Siemens data may perform better with our dataset, which may explain the narrower boxplots in the scan-rescan setting for AssemblyNet compared to AIRAscore or FastSurfer and the relatively small impact of scanner on absolute volumes. Including scanners from different manufacturers could yield different results for all tools; therefore, our results cannot be generalized to non-Siemens scanners^17^.

Our results indicate that deep learning tools such as AssemblyNet, AIRAscore, and FastSurfer demonstrate high reliability. In a previous study investigating the scan-rescan reliability of FreeSurfer, it was shown that the software had high reproducibility for total brain segmented volume regardless of scanners, head coils, or sequences^18^. Our findings align with these findings, showing no significant effect on volume measurements due to scanner-coil combinations. Another study that examined FreeSurfer (v7.3), FSL-FAST, CAT12, ANTs research tools for gray matter, white matter, and total intracranial volume found that all methods provided yielded consistent and reproducible measurements across subjects of well below 1%, though there was notable variability between methods^19^. Similarly, we observed greater variability between the methods we tested in our study. Moreover, our data expands on previous results by comparing the scan-rescan reliability of seven software solutions, including CE-labeled commercially available products and deep learning tools. It is important to emphasize that this study does not aim to support any tool but to highlight the significant issue of variability in volumetric analysis. We stress the importance of recognizing the variability introduced by different software and scanners over time, which must be considered when quantifying clinically relevant changes in brain volume.

This study has several limitations. One limitation is the lack of datasets from a broader range of subjects. A larger dataset would help in calculating correction factors for volumetric variability. To address this limitation, we used a homogenous set of participants and scan protocols; however, there were only a few datasets available for certain specific scanner software combinations, limiting statistical analysis. Future studies should aim to acquire more datasets from different subjects using the same software to build a broader database for calculating correction factors for volumetric variability. A higher number of subjects would increase the sample size for specific subgroup-combinations (scanner-software) and therefore result in statistically higher power. Also, we do not have any data for older patients yet. For this first study, we aimed for a very homogenous group of subjects. To reach more generalizable results, further studies with an increased number and more diverse subjects, and different scanner vendors are needed. Additionally, only T1-weighted scans were used in this study for volumetric analysis. It is possible to investigate the effects of other sequences, such as synthetic 3D T1 datasets derived from other imaging contrasts in future studies^20^. Due to availability, we only used scanners of one manufacturer. On the one hand this allowed a very homogenous design of the measurement protocols, on the other hand this is a limitation concerning generalizability of the results. Some software solutions (AssemblyNet) have been trained solely on Siemens data and might therefore perform better in this setting than on data of different scanners. It is also important to assess the impact of hydration status on volumetric measures, considering age and the influence of medications such as cortisone and antipsychotic drugs, which are known to affect brain volume^21^. The decision to focus on gray matter and white matter may have obscured differences in smaller brain volumes across software solutions and scanners. A previous study found that CV range between 1.6% for caudate and 6.1% for thalamus volume^22^. Different scanner/software combinations might produce varying volumetric results for different brain regions due to different anatomic definitions. However, by only evaluating total brain volume, gray matter volume and white matter volume this should be neglectable.

In conclusion, accurate volumetric measurements are essential for diagnosing and monitoring of neurodegenerative diseases, and planning therapy. New treatments for Alzheimer disease with anti-Aβ-antibodies can affect brain volume with unknown long-term effects^23^. Therefore, an accurate monitoring of the new therapy with brain volumetry can be an important part of follow-up controls to detect side effects of therapy as well as disease progression^24^. Our findings show that reproducibility of volumetric measurements varies significantly across software, with deep learning tools demonstrating higher reliability. As volumetric MRI analysis becomes more common, result interpretation must account for measurement protocol, scanner, software, and patient-specific factors (e.g., hydration status and medications). Establishing guidelines for correction factors would further improve the comparability of volumetric analysis, resulting in earlier, more accurate diagnoses and possibly improved treatment outcomes. Based on our results, we can recommend using the same combination of scanner and software across sessions to ensure that observed changes in brain volume are most reliable and clinically valuable.

## Methods

### Ethics approval and participants

The study was approved by the ethics committee at the medical faculty of the Eberhard Karls University and at the University Hospital of Tübingen (approval number: 512/2020BO). All experiments were conducted in accordance with ethics committee guidelines and regulations. Participants provided written, informed consent prior to the examination. Exclusion criteria included age below 18 or above 65, known structural anomalies of the brain, pregnancy, and contraindications for magnetic resonance imaging (MRI).

### Scanners and scanning protocol

Six different MRI scanners (all Siemens Healthineers, Erlangen, Germany) for imaging studies were used to acquire a T1-MPRAGE: three 1.5-Tesla scanners, including two distinct Aera scanners, located in separate rooms and referred to as Aera and Aera3, and one Avanto scanner, all equipped with 20-channel head-neck coils. Additionally, three 3-Tesla scanners were used including: Vida, Vida Fit, and Prisma, all fitted with 20-channel head-neck coils. For two 3 Tesla scanners (Prisma and Vida fit) scans with a 64-channel head-neck coil were acquired. For the 1.5 Tesla scanners, the scanning parameters were TR = 2400 ms, TI = 1000 ms, flip angle = 8°, bandwidth = 180 Hz/Px, 176 slices. For the 3 Tesla scanners, the scanning parameters were TR = 2300 ms, TI = 900 ms, flip angle = 9°, bandwidth = 240 Hz/Px, 176 slices. Scanning protocols were as described by Siemens Healthineers for the syngo.via evaluation.

### MRI preprocessing and volumetric software

All images were evaluated for visible motion artifacts. Registration to anatomical reference spaces, such as MNI or Talairach, was performed by each software where required. For the volumetric analyses, seven different software programs were used: FreeSurfer Version 7.1.1^25,26^, SPM12 version 7771^27^ running on Matlab 2018^28^ AIRAscore (Version 2.1.0, AIRAmed GmbH, Tübingen, Germany)^29^, AssemblyNet^30^, FastSurfer^31^, Vol2Brain^32^ and Brain Morphometry as part of the Neurology Workflow in syngo.via (VA40, Siemens Healthineers)^33^. FastSurfer and FreeSurfer do not provide a total WM label. Therefore, to create a total WM volume comparable to the other solutions, the output of the following labels was combined [Left|Right] Cerebellum-White-matter, Brainstem, [Left|Right]-VentralDC, [Left|Right]-Cerebral-White-Matter and corpus callosum labels (CC_Posterior, CC_Mid_Posterior, CC_Central, CC_Mid_Anterior, CC_Anterior). For FreeSurfer, SPM12, FastSurfer, and AssemblyNet DICOM raw data was converted into NIFTI-1 files with dcm2niix (version 1.0.20211006). For one dataset (Proband2_Avanto_Messung1), Vol2Brain and AssemblyNet failed segmentation, due to tilted head position. After manual correction to AC-PC alignment, the dataset could be segmented, and these values were used for further evaluations. AIRAscore and syngo.vio accepted DICOM images as input.

### Procedure

**Fig. 4:**
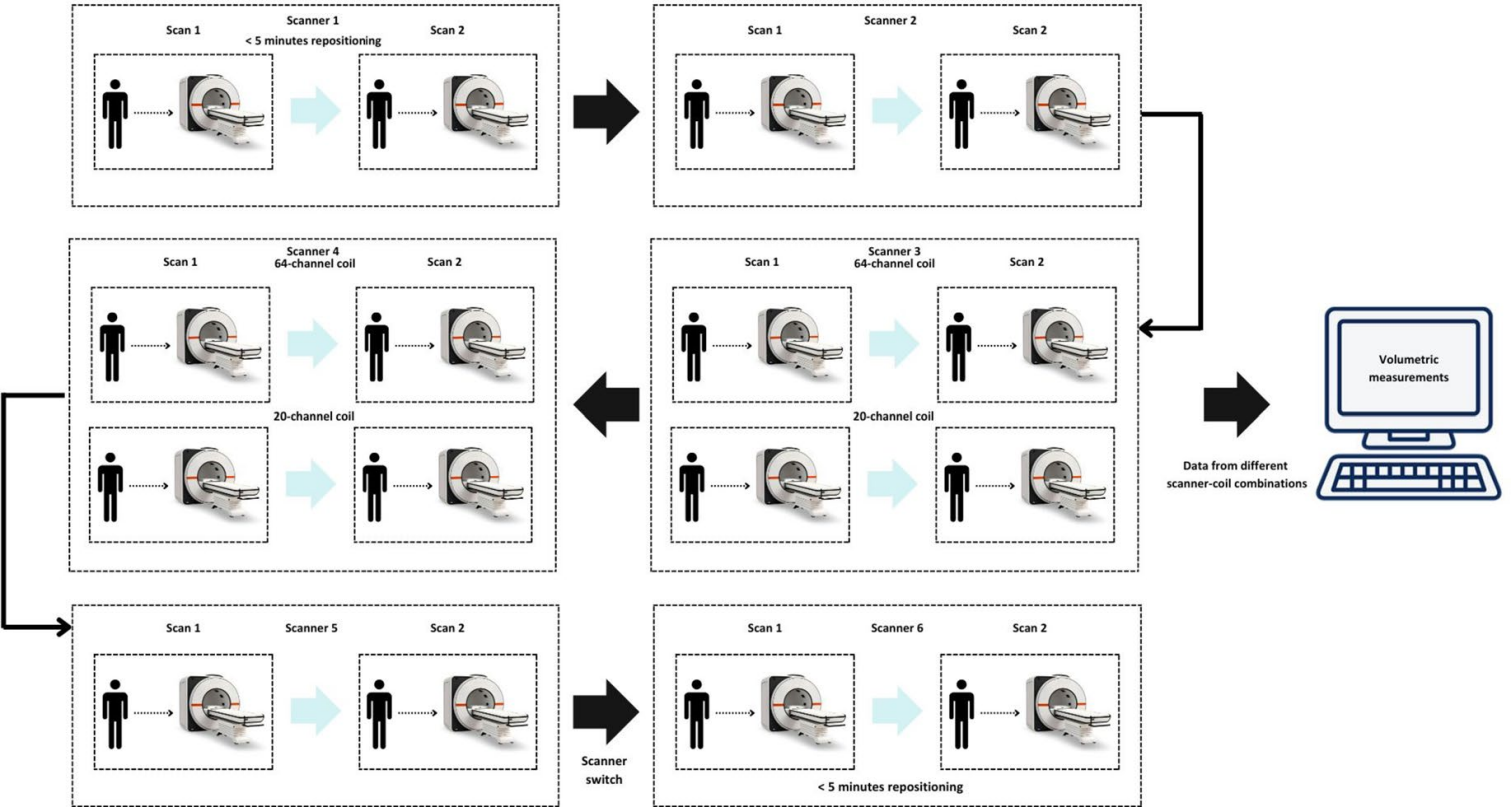
The figure illustrates the scanning procedure used to assess reliability of brain volumetric analysis across multiple scanner-coil combination and software. Six scanners were used (MAGNETOM: Aera, Aera3, Avanto, Prisma, Vida, Vida Fit). For each scan, a brain volumetric analysis was conducted using each software (SPM12, FreeSurfer, AIRAscore, AssemblyNet, Vol2Brain, FastSurfer, Syngo.via). Twelve subjects were scanned in each of the scanners. Each subject was scanned twice in every scanner on the same day. A third and fourth run was conducted on two 3 T scanners with an additional 64-channel head coil. Between each scan, subjects were moved out of the scanner, asked to reposition themselves, and then moved back into the same scanner. A localizer was acquired for each scan.

A prospective balanced design was used. Each participant was scanned twice using eight different combinations of scanner and coil on the same day, resulting in a total of 16 scans per participant, except for one participant who had only 12 scans due to fitting issues with the 64-channel coil. For the different scanners, there was a location switch. The total duration was approximately 2 h per participant (5 min per scanning session). Between each scan, the participant was moved out of the scanner, repositioned, and then moved back into the scanner. A localizer was acquired for each scan (see Fig. 4). The scanner image, computer image, and brain shape depicted in Fig. 4 were incorporated using elements from Canva.com.

### Statistical analysis

IBM Corp. Released 2023. IBM SPSS Statistics for Windows, Version 29.0.2.0 Armonk, NY: IBM Corp was used for statistical analysis. Python Version 3.10.12 was used for calculating the coefficient of variance. Statistical analysis was split into two parts. In the first the effect of software, scanner and session (first and second scan) on estimations of gray matter and white matter was evaluated using generalized estimation equations, while in the second step test-retest performance of each software was evaluated based on the general recommendation not to switch software or scanner for follow-up examinations.

### Statistical analysis using generalized Estimation equations

A generalized estimation equations (GEE) was computed to evaluate the effect of scanner, session (first or second scan with the same scanner), and software on the measured volume for gray matter and white matter. The dependency of the measurements due to the measurement of the same subject under different circumstances (scanner, software, session) was included in the linear model. The model was computed on the full dataset of the 12 subjects using the combination scanner and 20-/64- channel coil. For statistical comparison, AssemblyNet software and Prisma scanner were used as references. The Prisma scanner was used as the reference scanner because it is a widely recognized and commonly used standard in neuroimaging studies, providing a robust baseline for comparison. AssemblyNet was used as the reference software because it employs a novel deep learning approach combining two assemblies of U-Nets, each based on a large number of convolutional neural networks (125 CNNs) to achieve fine-grain segmentation of various anatomical regions, utilizing a training dataset of 45 manually segmented cases from the OASIS-dataset, which includes a diverse range of subjects and different Siemens scanners, ensuring high accuracy and generalizability in neuroimaging studies^34^.

### Measuring reproducibility of volume measurements

To measure the reproducibility of volume measurements for gray matter, white matter, and total brain volume (TBV) the percentage coefficient of variance $\:\text{\%}$ ($\:CV=\frac{\text{S}\text{D}}{\text{M}\text{e}\text{a}\text{n}}$ was calculated for all repeated measurements on the same scanner in the same subject. As CV follows a Chi-distribution median and interquartile range (IQR) were used to describe the results and boxplots and Bland-Altman-Plots were used for visualization to count the systematic volume differences between the different software or scanners the percentage change was used instead of absolute volume difference of repeated scans.

## Supplementary Information

Below is the link to the electronic supplementary material.

Supplementary Material 1

Supplementary Material 2

## Data Availability

Due to GDPR limitations raw imaging data may not be shared, the volumetric results of the different tools for each case is provided within the supplementary information files.
