# Supplementary material for: Scan-rescan reliability assessment of brain volumetric analysis across scanners and software solutions
Source: Sci Rep. 2025 Aug 14;15:29843. doi: 10.1038/s41598-025-15283-3 (PMC12354914; doi:10.1038/s41598-025-15283-3)
Supplement: Supplementary file 1 — Supplementary Material 1 [file 41598_2025_15283_MOESM1_ESM.pdf]

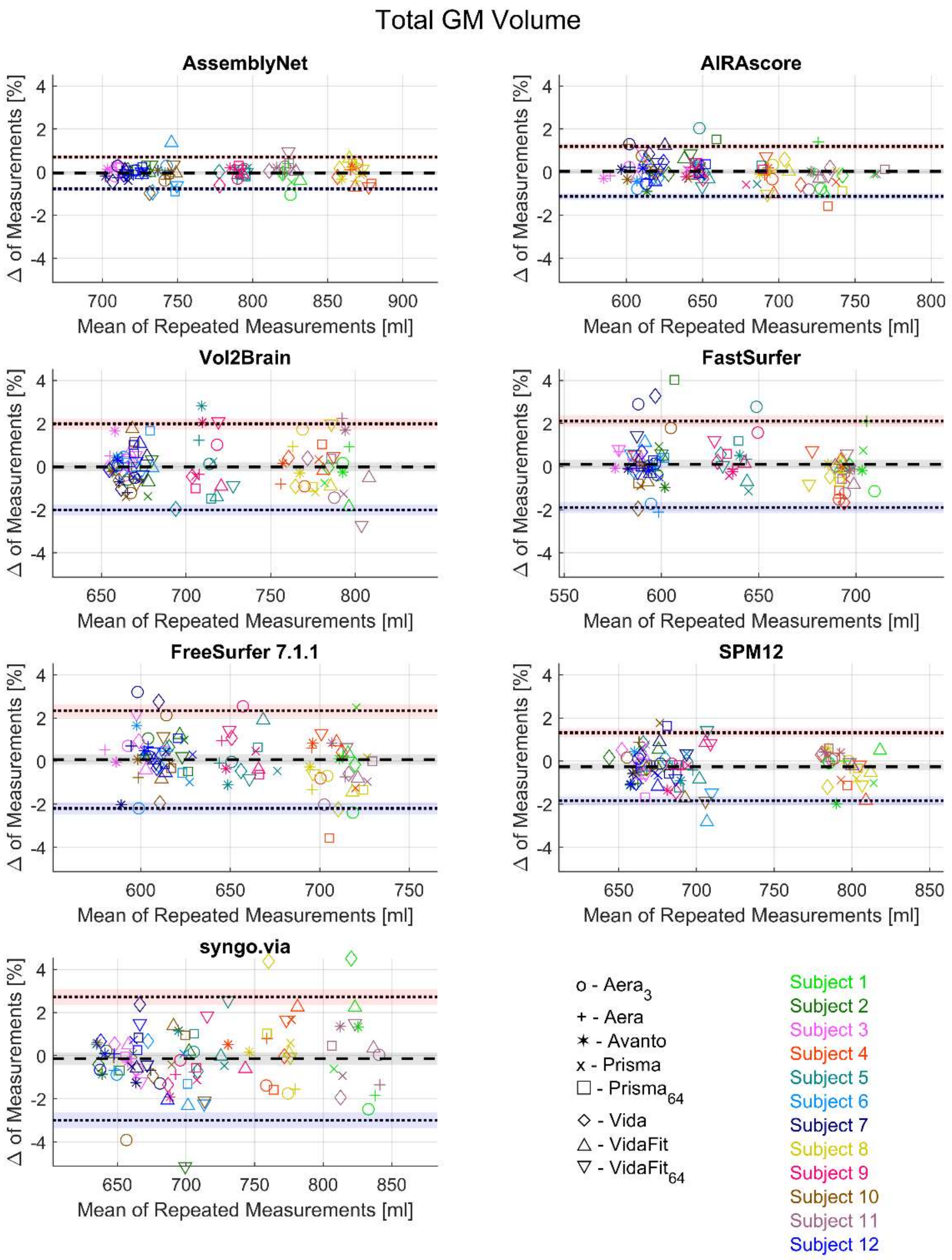

Supplementary material 1. Bland-Altman plot shows the comparison of total gray matter volume across different brain volumetry solutions. Each scanner is represented by a unique shape: circle for Aera 3, plus for Aera, star for Avanto, x-shape for Prisma, square Vida, triangle upward VidaFit, triangle downward VidaFit 64. Each subject is represented by a different color, allowing individual subject tracking across the comparisons. The x-axis represents the mean gray matter volume between the two sessions, while the y-axis displays the difference in percentage volume between the two sessions.

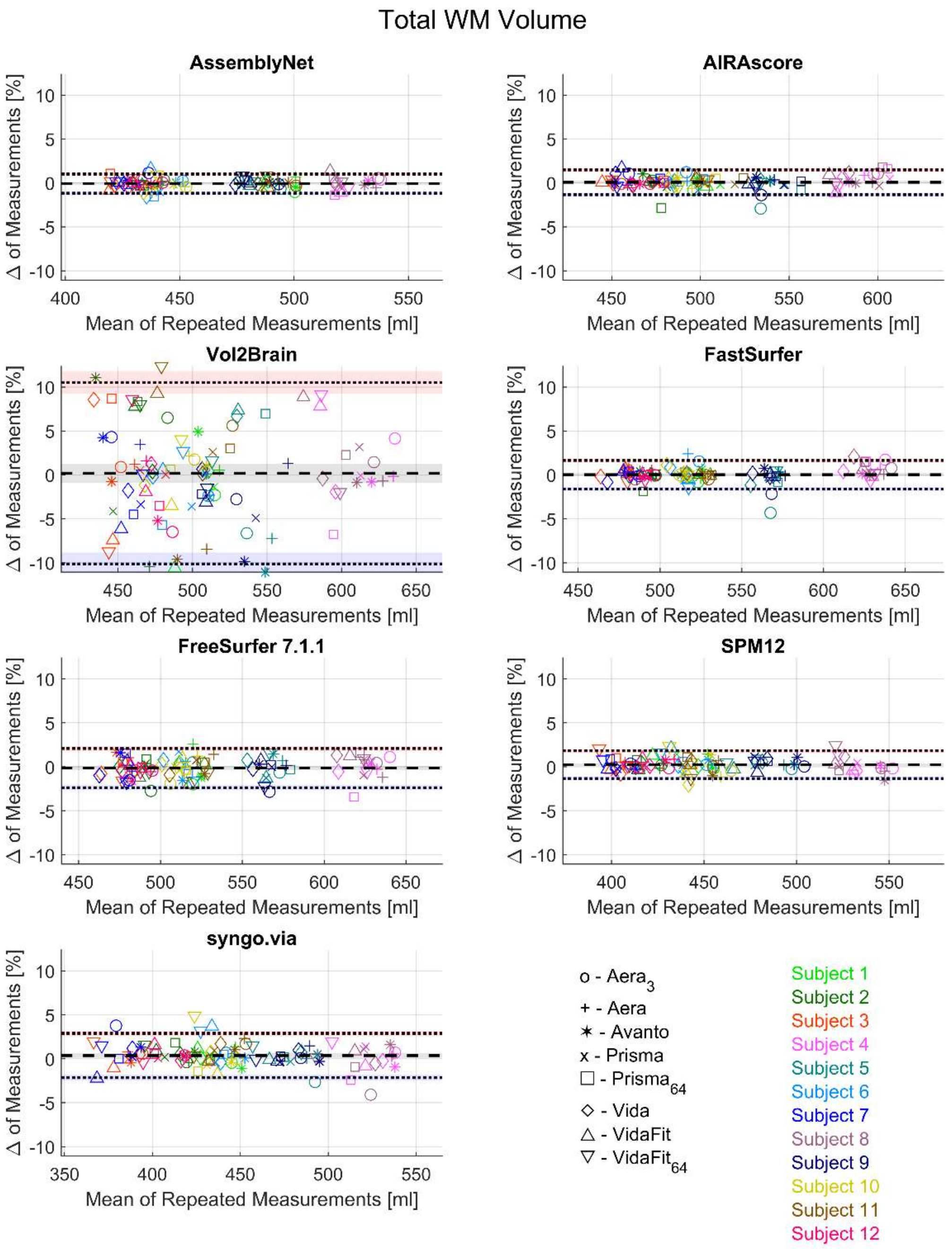

Supplementary material 2. Bland-Altman plot shows the comparison of total white matter volume across different brain volumetry solutions. Each scanner is represented by a unique shape: circle for Aera 3, plus for Aera, star for Avanto, x-shape for Prisma, square Vida, triangle upward VidaFit, triangle downward VidaFit 64. Each subject is represented by a different color, allowing individual subject tracking across the comparisons. The x-axis represents the mean white matter volume between the two sessions, while the y-axis displays the difference in percentage volume between the two sessions.

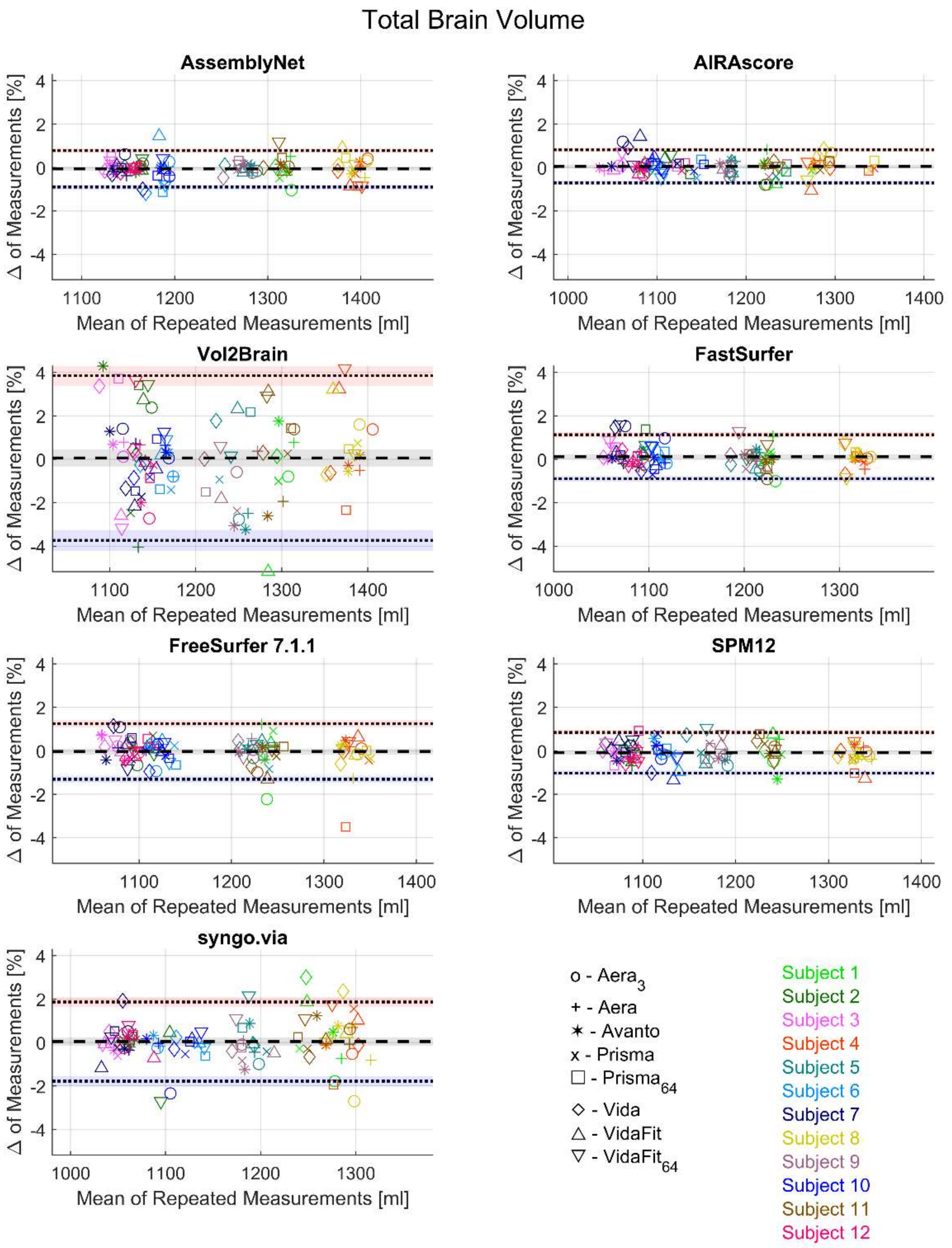

Supplementary material 3. Bland-Altman plot shows the comparison of total brain volume across different brain volumetry solutions. Each scanner is represented by a unique shape: circle for Aera 3, plus for Aera, star for Avanto, x-shape for Prisma, square Vida, triangle upward VidaFit, triangle downward VidaFit 64. Each subject is represented by a different color, allowing individual subject tracking across the comparisons. The x-axis represents the mean total brain volume between the two sessions, while the y-axis displays the difference in percentage volume between the two sessions.
